# Diplopia in Movement Disorders: A Systematic Review of the Literature

**DOI:** 10.3390/jpm14030270

**Published:** 2024-02-29

**Authors:** Larisa Ungureanu, Laura Irincu, Stefania Diaconu, Bianca Oprițoiu, K. Ray Chaudhuri, Cristian Falup-Pecurariu

**Affiliations:** 1 Department of Neurology, County Clinic Hospital, 500365 Brașov, Romania; 2 Faculty of Medicine, Transilvania University, 500036 Brașov, Romania; 3 Parkinson Foundation Centre of Excellence, King’s College, Denmark Hill Campus, King’s College Hospital, London SE5 9RT, UK; 4 Department Basic and Clinical Neuroscience, The Maurice Wohl Clinical Neuroscience Institute, King’s College London, Cutcombe Road, London SE5 9RT, UK

**Keywords:** diplopia, double vision, movement disorders, Parkinson’s disease, multiple system atrophy, progressive supranuclear palsy

## Abstract

Introduction: Although the reported frequency of diplopia is between 10 to 40% of patients with Parkinson’s disease (PD) and other movement disorders, it remains one of the most undiagnosed non-motor symptoms. Furthermore, it has a major impact on the quality of life of these patients. The aim of this study is to systematically review the literature regarding the frequency, causes, and implications of diplopia in movement disorders. Methodology: An electronic search was conducted in March and June 2023 using the PubMed database in order to identify appropriate studies. Studies that were written in English, that represented observational, analytical studies, and case reports, and that provided information regarding diplopia in movement disorders were included in the systematic review. Results: A total of 686 articles were identified out of which 43 met the inclusion criteria. The studies included in the systematic review ranged from descriptive studies (case reports and case series) to analytical–observational studies (cross-sectional studies, prospective and retrospective cohort studies, and case–control studies). In Parkinson’s disease, the incidence of diplopia ranged from 10 to 38%. In these patients, diplopia was linked to the presence of visual hallucinations and cognitive decline but also to convergence insufficiency and the presence of motor fluctuations. Cases of diplopia secondary to deep brain stimulation were also reported. Diplopia was associated with longer disease duration and worse motor and non-motor scores. Diplopia was also reported in other movement disorders such as multiple system atrophy (frequency as high as 18%) and progressive supranuclear palsy (frequency as high as 39%) and was associated with increased mortality and shorter duration in life span. Conclusions: Diplopia occurs in up to 38% of patients with movement disorders and has a negative impact on their health-related quality of life. Treating physicians should actively ask about diplopia and other ophthalmological symptoms, as many patients do not spontaneously report them. The pathophysiology of diplopia is complex, and it involves heterogeneous peripheral and central mechanisms. The management of these patients should involve a multidisciplinary team of health professionals in order to provide appropriate, tailored management.

## 1. Introduction

Diplopia, meaning the simultaneous perception of two images of a single object, is a frequent complaint in neurological disorders.

Parkinson’s disease (PD) is a neurodegenerative disorder characterized by a wide range of non-motor symptoms including visual disturbances, key determinants of quality of life in PD patients. Visual disturbances in PD patients include dry eyes, diplopia, decreased blink rates, blepharitis, blepharospasm, visual hallucinations, and convergence insufficiency [1]. Diplopia was reported in 20% of the patients included in a cohort of 545 patients of a study that followed the prevalence of non-motor symptoms in PD in an international setting [2]. The frequency of diplopia in PD is estimated to be 10–38% according to other studies [1,3,4,5]. The mechanisms of diplopia in PD are complex and include ophthalmological disorders, motor fluctuations, dysfunction in the oculomotor pathway, and the higher-level pathways of the visual system [6].

Diplopia has also been reported as a complaint in other movement disorders such as multiple system atrophy, progressive supranuclear palsy, and dystonia but its real prevalence is unknown.

The aim of this study is to systematically review the literature regarding the frequency, causes, and implications of diplopia in movement disorders in order to provide a comprehensive view of the importance of its recognition.

## 2. Materials and Methods

### 2.1. Identification and Eligibility Criteria

The protocol for this systematic review was conceived based on PRISMA 2020 Checklist. The study protocol was not pre-registered on the International Prospective Register of Systematic Reviews.

An extensive literature search was conducted using the PubMed database. The literature search was mostly conducted in March 2023 with an additional literature search carried out in June 2023. The search terms were “diplopia”, “double vision”, “Parkinson’s disease”, “Multiple system atrophy”, “Progressive supranuclear palsy”, “Dementia with Lewy bodies”, “Cortico-basal degeneration”, “Dystonia”, “Chorea”, “Huntington’s disease”, “Myoclonus”, “Tics and Tourette”, “Restless leg syndrome” and these were combined with Boolean Operator “AND”.

Inclusion criteria: the articles included in our systematic review had to meet three pre-defined criteria:

- The articles had to be published in English;
- The articles had to provide information about double vision/diplopia in movement disorders (Parkinson’s disease, multiple system atrophy, progressive supranuclear palsy, dementia with Lewy bodies, cortico-basal degeneration, dystonia, chorea, Huntington’s disease, Myoclonus, tics and Tourette, and restless leg syndrome);
- The articles had to be descriptive studies (case reports and case series) and observational, analytical studies (case–control, cross-sectional, and cohort studies) and had to be published online.

There was no restriction regarding the year of publication.

Articles published in a language other than English, that represented other types of studies outside the ones mentioned above (reviews, systematic reviews, etc.), studies that were not available in full-text, and studies that did not provide information regarding the frequency, characteristics of diplopia (causes, association with disease duration and stage, with the treatment of the disease or with the presence of other non-motor symptoms, impact on quality of life) were excluded from this systematic review.

### 2.2. Study Selection and Data Extraction

After duplicates were removed, the screening of the title and abstracts was performed by two independent reviewers (LI and LU), and then the full text of the remaining articles was assessed by the reviewers in order to meet the eligibility criteria mentioned above. Disagreements were resolved by consensus through discussion with the other authors. Furthermore, the reference lists of the identified articles were manually screened by the reviewers in order to identify other possible relevant articles.

Data extraction in a standardized sheet was jointly performed by LU and LI, and comprised of study identifiers (name, authors, year of publication), study type, participant characteristics (number of participants, age, sex, subgroup sizes), frequency data (frequency of diplopia in subgroups), characteristics and key clinical correlates of diplopia (disease duration, disease stage, treatment of the disease, cognitive dysfunction and other non-motor symptoms, quality of life).

## 3. Results

**Figure 1:**
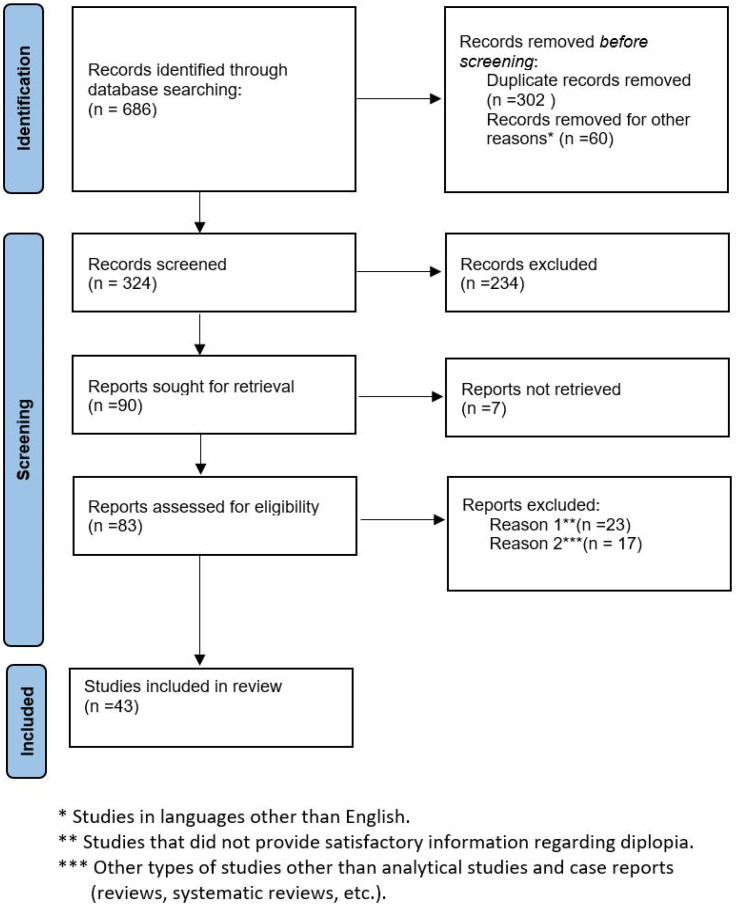
Flow chart of study selection process.

A total of 686 records were retrieved out of which 302 duplicates were removed. After applying the eligibility criteria, a total of 43 studies were included in this systematic review. The study selection process is shown in Figure 1.

### 3.1. Diplopia and Parkinson’s Disease

After the database search, we retrieved a total of 129 articles (out of which 51 duplicate articles were removed). The rest of the articles were manually screened by the authors in order to meet the eligibility criteria. In the end, a total of seventeen articles remained: nine were observational cross-sectional studies [3,4,6,7,8,9,10,11,12], two were prospective cohort studies [13,14], two were retrospective studies [15,16] and four were case reports [17,18,19,20]. Table 1 shows detailed information about the included studies on diplopia in Parkinson’s disease.

One study compared patients with PD, PD dementia, and healthy controls [11], two studies compared PD patients with diplopia, PD patients without diplopia, and healthy controls (HC) [12,13], four studies examined patients with PD compared to healthy controls [4,10,13,14], six studies examined PD patients with and without diplopia [3,6,7,8,15,16], and four were case reports of PD patients with diplopia [17,18,19,20].

The types of diplopia were selective diplopia (diplopia of a single object) or complete diplopia (diplopia of the entire visual field). Selective diplopia was associated with visual hallucinations in PD patients [8,12].

The frequency of diplopia in these studies ranged from 11.2% to 38%.

In PD patients, diplopia was associated with longer disease duration, higher doses of levodopa therapy, and more advanced Hoehn–Yahr stages. Furthermore, cognitive problems, apathy, and a higher frequency of motor fluctuations were significantly more often reported in PD patients with diplopia compared to PD patients without diplopia [6,9].

Mechanisms of diplopia are heterogenous and include convergence insufficiency [6,15,17], strabismus, ocular pathology as well as other mechanisms related to motor fluctuations [18], the presence of visual hallucinations [3,4,6,7,8,12,13], and the treatment of Parkinson’s disease [8,19,20].

### 3.2. Diplopia and Other Movement Disorders

After the database search of diplopia/double vision and each movement disorder, excluding Parkinson’s disease (Table 1), we retrieved a total of 557 articles (out of which 251 duplicates were removed). The rest of the articles were manually screened by the authors in order to meet the eligibility criteria. In the end, a total of 26 articles remained: five were retrospective studies [21,22,23,24,25], three were prospective studies [26,27,28], nine were case reports [29,30,31,32,33,34,35,36,37], six were case series [38,39,40,41,42,43], and three were observational cross-sectional studies [44,45,46]. Table 2 shows detailed information about the included studies on diplopia in other movement disorders (besides Parkinson’s disease).

**Hypokinetic movement disorders**

*Diplopia and Multiple system atrophy (MSA*)

Diplopia was reported as an ocular manifestation of MSA in two studies by Garcia M.D. et al. [21] and Sakakibara R. et al. [29], respectively. Both peripheral as well as central mechanisms were implicated in the appearance of diplopia in these patients.

*Diplopia and Progressive supranuclear palsy (PSP)*

In PSP, diplopia was reported as a complaint in patients diagnosed with wall-eyed bilateral internuclear ophthalmoplegia (WEBINO) in two studies by Yazdi N. et al. [30] and Matsumoto H. et al. [31], respectively. By studying a cohort of 187 patients with a possible or probable diagnosis of PSP, Nath U. et al. found that diplopia was present in 39% of cases (74 patients) [44].

*Diplopia and Corticobasal degeneration (CBD)*

Diplopia as an ocular manifestation of CBD was reported in a single study by Moura J. et al. [33].

**Hyperkinetic movement disorders**

*Diplopia and Dystonia*

In blepharospasm, a focal dystonia, diplopia has been reported as a frequent side effect of botulinum toxin injections. In all cases, the incidence of diplopia was between 2 and 11% and the symptomatology was spontaneously resolved in a short period of time [22,23,24,26,27]. Other studies have postulated that diplopia is secondary to ocular movement abnormalities that may accompany blepharospasm [38] or convergence spasm [25]. Congenital or genetic abnormalities have also been reported to cause diplopia in patients with dystonic disorders [39,40].

*Diplopia and Chorea*

Diplopia as an ocular manifestation in chorea was reported in a single study published in 1972 by Schieken R. et al. who described two cases of children who developed diplopia in the context of acute rheumatic fever [41].

*Diplopia and Tremor*

Diplopia is a frequent non-motor symptom in PD patients but not in patients with essential tremor (ET), as shown in a study by Giorelli et al. [45]. However, diplopia can be secondary to thalamic deep brain stimulation as a means of treatment for ET [46].

*Diplopia and Tourette syndrome*

Diplopia was reported as a secondary effect of thalamic deep brain stimulation for Tourette syndrome in a study by Ackermans L. et al. [34].

*Diplopia and Stiff-Person Syndrome (SPS)*

Several case reports and case studies mentioned diplopia as a frequent ocular manifestation of SPS [28,35,42,43]. Causes of diplopia in these patients include muscle spasms, skew deviation, or the presence of cranial nerve palsies and nystagmus [35].

*Diplopia and Myoclonus*

Kabanovski et al. [36] and Danesh-Meyer HV [37] described the cases of two patients who presented sudden onset diplopia and oculopalatal myoclonus due to the presence of pontine lesions.

## 4. Discussion

### 4.1. Hypokinetic Movement Disorders

#### 4.1.1. Pathophysiology, Frequency, and Implications of Diplopia in PD

Diplopia is a frequent complaint in PD [1,2,3,4,5]. The mechanisms that lead to the appearance of diplopia in PD are complex. Basal ganglia dysfunction is thought to cause excessive inhibition of the superior colliculus (an important structure that contributes to smooth eye movements, saccades, and vergence movements), thus leading to reduced amplitude of eye movements in PD patients [12]. Dysfunction of the control centers of ocular movement of the brainstem, subcortical, and cortical motor pathways in the visual parietal lobe, occipital, and temporal lobes are related to the presence of both transient and formed hallucinations that have been associated with diplopia in PD patients [6,47].

The frequency of diplopia in PD patients may vary between 10 and 38%, although PD patients may not report diplopia unless specifically asked about it [1,3,4,5]. An international study found diplopia to be undeclared by PD patients in 33.2% of cases [48]. Visser F. et al. reported in their study that 40% of the patients complained daily of diplopia, while 60% of the patients only reported this symptom weekly [12]. Schindlbeck K.A. et al. reported that diplopia was disclosed by 29.6% of the patients (37 out of 125 patients with PD) [6] while Archibald N.K. et al. found an incidence of diplopia of 38% in both the PD and PD dementia group [11]. In a study by van der Lijn I. et al. that aimed to determine the prevalence of visual complaints in 581 PD and 583 HC patients, the prevalence of diplopia reported “often/always” by PD patients was 10.8% compared to only 2.1% in the HC group. The total prevalence of diplopia was 11.2% in the PD group [10].

Types of diplopia found in PD patients include binocular diplopia (of both eyes), monocular diplopia (of one eye), and selective diplopia (duplication of specific objects). Convergence insufficiency and decompensated strabismus lead to binocular diplopia while ophthalmologic disorders such as refractive errors, lens opacities, macular or corneal disease, and tear film abnormalities can lead to monocular diplopia. Convergence insufficiency is a frequent cause of diplopia. This was present in 55.6% vs. 11.1% of patients with PD and diplopia compared to those without [6]. In 29 out of 44 PD patients with diplopia, diplopia could be attributed exclusively to convergence insufficiency in a study by Lepore F. [15]. Racette B.A. et al. reported the case of a patient with idiopathic PD who developed horizontal diplopia due to convergence insufficiency that appeared only during his off motor periods, nine years after being diagnosed with the disease. In this case, the convergence insufficiency was responsive to levodopa treatment [17].

Furthermore, selective diplopia was associated with visual hallucinations and cognitive dysfunction in PD patients [3,7,8,11,12] and is believed to be a part of a continuum of symptoms related to the PD psychosis spectrum that also includes visual illusions, hallucinations, and delusions [9]. Archibald N.K. et al. described the spectrum of visual symptoms of Parkinson’s disease in a group of 64 subjects with Parkinson’s disease, 26 subjects with Parkinson’s disease and PD dementia (PDD), and 32 age-matched healthy controls using a thorough ophthalmological, neuropsychological, and diagnostic assessment. They found that the duration of the disease, Epworth Sleepiness Scale (ESS) scores, abnormal ocular alignment, and hypometric saccades were all predictive factors for the presence of diplopia. Furthermore, visual symptoms were more commonly found in the PD dementia group compared to the non-dementia PD patients [11]. Visual hallucinations were found in 44% of the PD patients with diplopia in a study by Visser et al., with 13% of the patients reporting the simultaneous presence of both diplopia and visual hallucinations [12]. Nebe et al. reported that nine out of fourteen patients with selective diplopia had visual hallucinations while three more patients developed visual hallucinations in the following 3 years from the onset of diplopia [8]. Visual hallucinations were present in 45.7% vs. 9.4% of patients with PD and diplopia compared to those without diplopia at the first visit and in 53.5% vs. 13% at the third visit, in a 2-year follow-up study by Santos García D. et al. [13]. A total of 19.2% of PD patients with diplopia reported the co-occurrence of diplopia with visual hallucinations [6] while nine patients reported diplopia as a type of visual illusion in a study by Sasaki C. et al. [7]. Visual hallucinations in PD were associated with difficulty perceiving spatial relations and double vision [3]. Urwyler P. et al. reported that diplopia and misjudging objects are predictors for passage hallucinations in patients with PD [4].

Diplopia has also been linked to motor “off” periods and motor fluctuations in some patients [17,18] and to the treatment of the disease [8,19,20]. Likitgorn T. et al. reported the case of a patient with an 8-year history of PD who developed diplopia. The patient exhibited intermittent severe difficulty with saccade initiation and freezing of saccades that were variable and occurred suddenly during the examination. Smooth pursuit could interrupt the freezing of reflexive saccades and the freezing dramatically resolved after the increase in levodopa-carbidopa dosage [18]. In the study by Nebe et al., it was reported that diplopia was preceded by the administration of amantadine or by up-titration of dopamine agonists or levodopa (three and four patients, respectively), while deep brain stimulation (DBS) led to the appearance of diplopia in one patient. Furthermore, the administration of quetiapine or clozapine led to the disappearance of diplopia in three patients [8]. Diplopia was also associated with DBS for advanced Parkinson’s disease in two case reports by Ortiz-Pérez et al. [19] and Miyagi et al., respectively [20].

In PD, the spectrum of non-motor symptoms (psychiatric symptoms, cognitive impairment, pain, fatigue, sleep disturbances, etc.), have been shown to be major determinants of patients’ worsening health-related quality of life (HRQoL) [49]. Patients with diplopia frequently complain of visual disorientation and difficulties with daily activities such as walking and reading [50]. Patients with PD and diplopia had worse motor and non-motor status as well as a higher dependency on activities of daily living (ADL) compared to those without diplopia [13]. Hamedani A.L. et al. found that diplopia in PD is associated with longer disease duration, hallucinations, higher scores at MDS UPDRSS part II and NMSQuest as well as with other indicators of disease severity [14]. Thus, diplopia is an important factor that further affects the quality of life of patients with Parkinson’s disease.

#### 4.1.2. Frequency and Implications of Diplopia in MSA

Garcia M.D. et al. studied the ocular manifestations of MSA in a cohort of 19 cases of MSA-P and 20 cases of MSA-C. Ocular misalignment leading to diplopia was present in seven out of the thirty-nine patients (18%), six of whom were in the MSA-C group. Furthermore, dry eye leading to symptomatic monocular diplopia was present in five cases, while trichiasis due to ocular cicatricial pemphigoid leading to diplopia was present in one patient. The authors found that MSA patients with ocular findings (excluding dry eye) had a significantly shorter duration of life compared to MSA patients without ocular findings [21]. Vergence paresis led to symptomatic diplopia in two MSA patients in a study by Sakakibara R. et al. [29].

#### 4.1.3. Frequency and Implications of Diplopia in PSP

Yazdi N. et al. presented the case of a 57-year-old man with a history of progressive gait disturbances who developed binocular nonfluctuating horizontal diplopia. Clinical examination revealed a severe limitation of vertical eye movement, bilateral exotropia in the primary position, and severely limited adduction of both eyes with nystagmus on the abducting eye, while brain IRM revealed a typical humming bird sign, without any other lesions that could explain the ocular abnormalities. The authors concluded the case to be a wall-eyed bilateral internuclear ophthalmoplegia (WEBINO) as an ocular finding in PSP [30]. This is in concordance with a case described by Matsumoto H. et al. of a 72-year-old man with identical findings, concluding that the lesions of the bilateral medial longitudinal fasciculus and the overexcitation of paramedian pontine reticular formation are the cause of WEBINO syndrome in patients with PSP [31].

Lowrey BR and Wong L described the case of a 69-year-old male with a history of imbalance and falls who presented frequent diplopia, both horizontal and vertical, as well as transient blurry vision. Clinical examination revealed a vertical gaze deficiency, hypometria in all fields of gaze as well as a marked decreased blink rate and incomplete lid closure. The findings were consistent with the diagnosis of PSP [32].

Early speech or swallowing problems and diplopia were associated with increased mortality in a large cohort study by Nath U. et al. [44].

#### 4.1.4. Diplopia in CBD

Moura J. et al. described the case of a 51-year-old male with neuropathological confirmed rapid progressive CBD. He presented with diplopia, bilateral ptosis, and dysphagia and rapidly developed cognitive dysfunction, asymmetrical parkinsonism, and gaze limitation. The differential diagnosis included PSP and immune-mediated brainstem encephalitis due to the clinical and paraclinical findings [33].

### 4.2. Hyperkinetic Movement Disorders

#### 4.2.1. Frequency and Implications of Diplopia in Dystonia

Botulinum toxin injections are frequently used in the treatment of blepharospasm, a focal dystonia, and a frequently encountered side effect of this procedure is diplopia. In 1988 Grandas F. et al. reviewed 264 patients with blepharospasm, 151 out of which were treated with botulinum toxin injections. They found transient diplopia as a side effect of the treatment in 17 out of the 151 patients [22]. At the same time, Dutton J.J. and Buckley E.G. evaluated the long-term outcomes and complications of botulinum toxin in 232 patients with blepharospasm and found that diplopia involving the inferior oblique or lateral rectus muscles is present in less than 1% of the group (five patients), and resolved spontaneously in 1 to 4 weeks [26]. Four out of twenty patients with blepharospasm treated with botulinum toxin A injections reported transient diplopia after treatment in a study by Berardelli et al. [27]. Martinez-Ramirez D. et al. compared the outcomes of botulinum toxin treatment between primary and secondary blepharospasm in a group of 64 subjects. Diplopia was the second most common side effect of the botulinum toxin treatment, with an incidence of 6% [23]. Jochim A. et al. studied the long-term safety and efficiency in daily clinical practice of abo- and onabotulinumtoxin A treatment for blepharospasm and Meige’s syndrome. Diplopia was reported in 2% of the patients treated with abobotulinumtoxinA and, respectively, in 2% of the patients treated with onabotulinumtoxinA [24].

Blepharospasm can be accompanied by ocular abnormalities. Aramideh M. et al. reported the cases of four patients with blepharospasm and eye movement disorders characterized by an inability to look fixedly and short or prolonged episodes of uncontrollable eye deviations and diplopia in horizontal or vertical directions (in two out of four patients). They concluded that various ocular movement abnormalities can accompany blepharospasm, not pointing necessarily to symptomatic dystonia [38]. On the contrary, another study presented the cases of 17 patients with diplopia secondary to convergence spasm who were treated with botulinum toxin with variable alleviation of symptoms [25]. The authors concluded that in some cases, convergence spasm may represent a type of dystonia, suitable for treatment with botulinum toxin.

Congenital or genetic disorders may lead to the appearance of diplopia. Varrato J and Galetta S reported the case of a woman with a long history of torticollis for which she received botulinum toxin injections and who shortly after developed binocular vertical diplopia that improved after the effects of the injections wore off. In this case, the diplopia was secondary to a congenital fourth nerve palsy, discovered after the treatment with botulinum toxin, when she was not able to maintain a compensatory head tilt contralateral to the impaired trochlear nerve [39]. Defects of the Synaptojanin 1 (SYNJ1) gene have been associated with early-onset atypical parkinsonism and neurodegeneration. Hong D. et al. presented the cases of two siblings with a symptomatic triad of diplopia, dystonia (severe trunk dystonia and dystonic postures of the limb), and parkinsonism, symptoms that resolved after treatment with clonazepam. Genetic testing revealed novel variants in the SYNJ1 gene co-segregating in the family [40].

#### 4.2.2. Diplopia in Chorea

Unilateral choreiform eye movements leading to loss of fusion of images as a mechanism of diplopia development were reported in two children with acute rheumatic fever in a study by Schieken R. et al. The ocular manifestations subsided after a few weeks and did not reappear [41].

#### 4.2.3. Diplopia in Tremor

Giorelli M. et al. evaluated the range of non-motor symptoms in a group of PD patients compared to a group of patients with essential tremor (ET), all of whom underwent DAT scans. They found that PD patients reported diplopia in 22.5% of the cases compared to ET patients who did not report diplopia as a non-motor symptom. Furthermore, PD patients with diplopia had a greater denervation of the cauda than the PD patients without diplopia [45].

Twenty-three patients with essential tremors who underwent deep brain stimulation in the ventro-intermedius nucleus were evaluated in regard to their quality of life and activities of daily living. The authors found diplopia as a side-effect of thalamic stimulation to be present in one patient [46].

#### 4.2.4. Diplopia in Tourette Syndrome

A 39-year-old man with Tourette syndrome underwent thalamic deep brain stimulation (DBS). Postoperatively, he developed diplopia due to vertical gaze palsy. A postoperative CT scan revealed a hyperdense lesion at the distal end of the left electrode that corresponds with bilateral pretectal components including the rostral interstitial nucleus of the medial longitudinal fasciculus. The clinical signs and symptoms gradually improved over the course of six months. The authors concluded that a special focus on vertical eye movements, extreme accuracy, and caution are needed in the planning of trajectory electrode positioning for thalamic DBS [34].

#### 4.2.5. Diplopia in SPS

Diplopia is a frequent complaint in patients diagnosed with SPS. Smith J. and Storey H. [35] reported the case of a 57-year-old woman who presented with a 2-week history of sudden onset diplopia, blurred vision, and lower limb spasticity secondary to muscle spasms that led to difficulty in walking and stair climbing. Orthoptic investigation revealed a left inferior rectus weakness and later developed gaze-evoked nystagmus and laterally alternating skew deviation. She was referred to a neurologist who diagnosed her with stiff-person syndrome. Piquet A. et al. followed the cases of 17 patients with SPS with parkinsonism or cerebellar signs and positive glutamic acid decarboxylase (GAD65) antibodies. Ten out of seventeen patients (59%) reported visual disturbances including intermittent diplopia [28]. Mas N. et al. presented the case of a 33-year-old woman who presented with diplopia, dysphagia, and gait ataxia and later developed typical SPS symptoms. Glycine receptor antibodies (GlyR-ab) were positive and treatment with corticosteroids and intravenous immunoglobulin (IVIG) was effective [42]. Anti-Zic4 antibodies were identified in a man who presented with diplopia, muscle stiffness, and fluctuating eyelid ptosis. The authors concluded that anti-Zic4 antibodies could be involved in the pathogenesis of SPS [43].

#### 4.2.6. Diplopia in Myoclonus

Pontine lesions may lead to the appearance of diplopia and myoclonus. Danesh-Mayer H.V. [37] presented the case of a 57-year-old woman with horizontal diplopia due to oculopalatal myoclonus (OPM) and other ocular movement abnormalities with sudden onset after surgical repair of a ruptured posterior fossa aneurysm. A similar case of a 69-year-old man with a pontine hemorrhage that led to the sudden onset of diplopia in the context of skew deviation and OPM was reported by Kabanovski A. et al. [36].

### 4.3. Quantification and Management of Diplopia in Patients with Movement Disorders

Causes of diplopia in patients with movement disorders are multiple and the mechanisms of diplopia arise from the pathophysiology of the diseases but also from secondary conditions that these patients can associate with (ocular pathology, diabetes mellitus, cerebrovascular disease, endocrine dysfunction) and these mechanisms can be intricated. Thus, it is very important to obtain a thorough personal and family history, to perform a thorough neurological, ophthalmological, and general examination as well as paraclinical investigations to exclude the presence of cerebrovascular disease, neoplasia, infections, autoimmune, and inherited diseases.

Diplopia can be quantified both subjectively and objectively. Holmes J.M. et al. [51] developed a scoring algorithm for their diplopia questionnaire, rating diplopia symptoms from 0 (no diplopia) to 100 (diplopia always and everywhere). Its advantage is that it does not require any specific equipment, is easy to administer, and reflects the patients’ experience. However, to our knowledge, specific diplopia questionnaires and scoring algorithms have not been validated for the PD population and other movement disorder patients, and their use may be limited by issues such as cognitive deficits, visual hallucinations, and severe motor “off” periods. Objective means that can evaluate ocular misalignment include the cover–uncover test, which is based on a fixational eye movement, meaning that in cases of ocular misalignment, one eye fixates the target while the other eye deviates, or the Maddox rod test, which uses transparent red plastic cylinders and a light source to create an optical phenomenon that allows the examiner to assess misalignment both in horizontal and in vertical planes. These are important tools with which we can quantify convergence insufficiency and strabismus, frequent causes of diplopia in PD patients [6,12,15], as well as patients with other movement disorders.

Management of diplopia includes specific ophthalmologic treatments for macular or corneal defects, cataract removal, and refraction correction. If the cause of diplopia is convergence insufficiency, an ophthalmologist or orthoptist can help prescribe single-vision reading glasses, base-in prisms, or perform monocular occlusion. The successful correction of diplopia with prisms was associated with improvement in HRQOL in patients with strabismus, specifically regarding their reading and overall function [52].

Also, typoscopes, e-tablets, and proper lighting to facilitate reading may help alleviate symptoms. Optimization of levodopa treatment in PD patients may lead to improvement or even disappearance of diplopia. If visual hallucinations are found to be the cause of diplopia, their treatment should be implemented.

**Limitations of present study and future perspectives**

Our study has some limitations. The main limitation of this systematic review comprises the heterogeneity of the studies that were included, in terms of design (case reports and cross-sectional studies), patient selection, outcome measures, and results. These aspects led to a difficulty in developing a meta-analysis of the studies included in the systematic review. Secondly, the systematic review was based on a search using only the PubMed database. Thirdly, another source of bias could be the language restriction, as we only included in this systematic review articles that were published in English, thus possibly missing some results.

Our study identified the presence and characteristics of patients with diplopia, mainly in small cohorts of patients or in individual reported cases. Future larger-scale studies are needed in order to fully comprehend the true prevalence and pathogenesis of diplopia in patients with movement disorders.

## 5. Conclusions

Diplopia is a frequent complaint and has a negative impact on the health-related quality of life of patients with movement disorders. Treating physicians should actively ask about diplopia and other ophthalmologic symptoms, as many patients do not spontaneously report them. The pathophysiology of diplopia is complex, and it involves heterogeneous peripheral and central mechanisms. The management of these patients should involve a multidisciplinary team of health professionals in order to provide appropriate, tailored care.

## Figures and Tables

**Table 1:** Included studies on diplopia in Parkinson’s disease (hypokinetic).

Author	Year of Publication	Type of Study	Number of Patients	Frequency of Diplopia	Mean Age (Years)	Male Gender	Disease Duration (Years)	H-Y Stage (Mean)	UPDRS Motor Score (Mean)	NMSS Score (Mean)	MMSE/MoCA Score (Mean)
Racette B.A. et al. [17]	1999	Case report	1 PD patient	n = 1	47	n = 1	10	NA	NA	NA	NA
Davidsdottir S. et al. [3]	2004	Observational cross-sectional study	81 PD patients	38%	63.8 ± 10.3	n = 56 (69%)	7.2 ± 4.8	2 (1–3)	NA	NA	28.7 ± 1.1
Lepore F. [15]	2006	Retrospective study	44 PD patients w/diplopia	n = 44	68.2 ± 8.8 (PD w/diplopia)	n = 28	8.3 ± 5.8 (PD w/diplopia)	NA	NA	NA	NA
			9 PD patients w/o diplopia		63.8 ± 9.9 PD w/o diplopia		4.3 ± 3.7 PD w/o diplopia				
Nebe A., Ebersbach G. [8]	2007	Observational cross-sectional study	14 PD patients	n = 14	67	n = 6	14	3 (2–5)	NA	NA	<24 (N = 6)
Ortiz-Pérez S. et al. [19]	2009	Case report	1 PD patient	n = 1	57	n = 1	8	NA	NA	NA	NA
Archibald N.K. et al. [11]	2011	Observational cross-sectional study	64 PD patients	n = 34 PD + PDD (38%)	70.2 ± 8.1 (PD)	66% (PD)	8.4 ± 5.7 (PD)	NA	23.1 ± 10.0 (PD)	NA	28.9 (PD)
			26 PD dementia patients		71.2 ± 6.5 (PDD)	84% (PDD)	11.5 ± 5.8 (PDD)		35.4 ± 14.7 (PDD)		23.6 (PDD)
			32 HC		72.2 ± 7.7 (HC)	47% (HC)	-		-		29.5 (HC)
Urwyler P. et al. [4]	2013	Observational cross-sectional study	88 PD patients	n = 16 (18.2%) PD	72.3 ± 9.1 (PD)	n = 47 (PD)	8.6 ± 5.6	NA	22.5 ± 10.6	NA	27.6 ± 2.7 (PD)
			90 HC	n = 1 (1.3%) HC	73.5 ± 8.4 (HC)	n = 38 (HC)	-		-		28.6 ± 1.9 (HC)
Schindlbeck K.A. et al. [6]	2017	Observational cross-sectional study	125 PD patients	n = 37 (29.6%)	70.5 ± 9.3 (PD w/diplopia)	n = 24 (PD w/diplopia)	10.1 ± 5.5 (PD w/diplopia)	3.0 ± 0.9 (PD w/diplopia)	36.4 ± 12.1 (PD w/diplopia)	NA	27.7 ± 1.5 (PD w/diplopia)
					73.3 ± 8.0 (PD w/o diplopia)	n = 41 (PD w/o diplopia)	6.4 ± 7.3 (PD w/o diplopia)	2.5 ± 0.7 (PD w/o diplopia)	31.5 ± 10.3 (PD w/o diplopia)		27.9 ± 1.6 (PD w/o diplopia)
Visser F. et al. [12]	2019	Observational cross-sectional study	25 PD patients w/diplopia	n = 25	72 (PD w/diplopia)	n = 18 (PD w/diplopia)	6 (PD w/diplopia)	3 (PD w/diplopia)	24 (PD w/diplopia)	NA	23 (PD w/diplopia)
			16 PD patients w/o diplopia		73 (PD w/o diplopia)	n = 10 (PD w/o diplopia)	6 (PD w/o diplopia)	2 (PD w/o diplopia)	19 (PD w/o diplopia)		23 (PD w/o diplopia)
			23 HC		70 (HC)	n = 12 (HC)	-	-	-		28 (HC)
Hamedani A.G. et al. [14]	2021	Prospective cohort study	26,790 PD patients	18.1% PD	67 (PD)	n = 14,867 (PD)	3.6	NA	25.2 ± 8.34	NA	NA
			9257 HC	6.3% HC	59.9 (HC)	n = 2178 (HC)	-		-		
Santos García D. et al. [13]	2021	Prospective cohort study	At baseline:	At baseline:	At baseline:	At baseline:	At baseline:	NA	At baseline:	At baseline:	NA
			691 PD patients	n = 94 (13.6%) PD	63.69 ± 7.73 (PD w/diplopia)	59% (PD w/diplopia) 59.2% (PD w/o diplopia)	6.12 ± 4.35 (PD w/diplopia) 5.27 ± 4.21 (PD w/o diplopia)		24.98 ± 10.93 (PD w/diplopia) 21.48 ± 10.36 (PD w/o diplopia)	65.33 ± 47.7 (PD w/diplopia) 38.14 ± 31.22 (PD w/o diplopia)	
			206 HC	n = 4(1.9%)HC	62.26 ± 8.78 (PD w/o diplopia)						
Naumann W. et al. [9]	2021	Observational cross-sectional study	24 PD patients w/diplopia	n = 24	74.3 ± 8.0 (PD w/diplopia)	n = 18 (PD w/diplopia)	8.5 ± 6.2 (PD w/diplopia)	NA	35.8 ± 11.8 (PD w/diplopia)	NA	NA
			26 PD patients w/o diplopia		70.4 ± 8.8 (PD w/o diplopia)	n = 14 (PD w/o diplopia)	7.4 ± 6.5 (PD w/o diplopia)		31.1 ± 10.2 (PD w/o diplopia)		
			24 HC		69.7 ± 9.1 (HC)	n = 7 (HC)	-		-		
Sasaki C. et al. [7]	2021	Observational cross-sectional study	40 PD patients	n = 9	64.4 ± 5.0	n = 21	7.0 ± 3.6	2.5 (1–4)	45.4	NA	NA
Likitgorn T. et al. [18]	2021	Case report	1 PD patient	n = 1	68	n = 0	8 years	NA	NA	NA	NA
Miyagi Y., Urasaki E. [20]	2021	Case report	1 PD patient	n = 1	61	n = 0	18 years	NA	NA	NA	NA
Kwan K. et al. [16]	2022	Retrospective study	43 PD patients	n = 16 (37%)	70 ± 8.9	n = 7	4.5 ± 4.5	NA	NA	NA	NA
Van der Lijn I. et al. [10]	2023	Observational cross-sectional study	581 PD patients	n = 65 (11.2%)	69.25 ± 9.01 (PD)	60.9% (PD)	7.96 ± 6.59	NA	NA	NA	NA
			583 HC	2.1%	69.17 ± 8.99 (HC)	63.3% (HC)	-				

**Table 2:** Included studies on diplopia in other movement disorders (excluding Parkinson’s disease).

Author	Year of Publication	Type of Movement Disorder	Movement Disorder	Type of Study	Number of Patients	Frequency of Diplopia	Mean Age (Years)	Male Gender	Disease Duration (Years)	Causes of Diplopia	Implications
Garcia M.D. et al. [21]	2017	Hypokinetic	Multiple system atrophy	Retrospective study	19 MSA-P patients	33% (n = 13 patients)	63.7 years (age at onset)	59% (n = 23)	8.9 (symptom onset to death)	Dry eyes—5 patients (monocular)	Shorter lifespan
					20 MSA-C patients		68.6 years (age at death)			Ocular misalignment—7 patients (6 MSA-C, 1 MSA-P)	
										Cicatricial pemphigoid with trichiasis—1 patient	
Sakakibara R. et al. [29]	2005	Hypokinetic	Multiple system atrophy	Case series	2 MSA patients	n = 2	79 (case 1)	n = 2	5	Vergence paresis (divergence)	NA
							68 (case 2)			Vergence paresis (convergence)	
Yazdi N. et al. [30]	2019	Hypokinetic	Progressive supranuclear palsy	Case report	1 PSP patient	n = 1	57	n = 1	5	Wall-eyed bilateral internuclear ophthalmoplegia (WEBINO)	NA
Matsumoto H. et al. [31]	2008	Hypokinetic	Progressive supranuclear palsy	Case report	1 PSP patient	n = 1	72	n = 1	6	Wall-eyed bilateral internuclear ophthalmoplegia (WEBINO)	NA
Lowrey BR, Wong L [32]	2000	Hypokinetic	Progressive supranuclear palsy	Case report	1 PSP patient	n = 1	69	n = 1	2	Gaze palsy	NA
Nath U. et al. [44]	2002	Hypokinetic	Progressive supranuclear palsy	Observational cross-sectional study	187 PSP patients	39% (n = 74 patients)	NA	48% (n = 89)	5.7 (deceased cases)	Gaze palsy	Diplopia is second earliest disease feature (after falls)
									6.4 (surviving cases)		Increased relative mortality
Moura J. et al. [33]	2022	Hypokinetic	Corticobasal degeneration	Case report	1 CBD patient	n = 1	51	n = 1	3	Gaze palsy	NA
Grandas F. et al. [22]	1988	Hyperkinetic	Dystonia	Retrospective study	264 patients with blepharospasm	11.3% (n = 17 patients)	55.8 ± 12.5 (age at onset)	35.6% (n = 94)	NA	Secondary to botulinum toxin injections (transient, 1–45 days duration)	NA
Dutton J.J. and Buckley E.G. [26]	1988	Hyperkinetic	Dystonia	Prospective cohort study	232 patients with facial dystonias	0.5% (n = 5 patients)	62.2	28% (n = 65)	NA	Secondary to botulinum toxin injections (transient, 1–4 weeks duration)	NA
Berardelli et al. [27]	1990	Hyperkinetic	Dystonia	Prospective cohort study	20 patients with blepharospasm	20% (n = 4) patients with blepharospasm	57.9 (blepharospasm)	45% (n = 9) blepharospasm	NA	Secondary to botulinum toxin injections	NA
					8 patients with torticollis		45.3 (torticollis)	75% (n = 6) torticollis			
					12 patients with hemifacial spasm		61.3 (hemifacial spasm)	25% (n = 3) hemifacial spasm			
Martinez-Ramirez D. et al. [23]	2014	Hyperkinetic	Dystonia	Retrospective study	64 patients with primary and secondary blepharospasm	6% (4 patients)	69.1 ± 9.2 (primary group)	32% (n = 13) primary group	14.2 ± 11.9 (primary group)	Secondary to botulinum toxin injections	NA
							65.9 ± 10.2 (secondary group)	74% (n = 17) secondary group	6.2 ± (secondary group)		
Jochim A. et al. [24]	2019	Hyperkinetic	Dystonia	Retrospective study	348 patients with blepharospasm and Meige’s syndrome	1% (ONA group)	56.3 ± 10.8 (age at onset, ONA group)	NA	NA	Secondary to botulinum toxin injections	NA
						1% ABO group	58.7 ± 10.3 (age at onset, ABO group)				
Aramideh M. et al. [38]	1994	Hyperkinetic	Dystonia	Case series	4 patients with blepharospasm and apraxia of eyelid opening	50% (n = 2)	50	n = 1	NA	Uncontrollable gaze deviations	NA
Kaczmarek B. et al. [25]	2009	Hyperkinetic	Dystonia	Retrospective study	17 patients with convergence spasm	n = 17	34 (age at treatment)	35% (n = 6 patients)	NA	Convergence spasm, alleviation of symptoms after botulinum toxin injections (in 10/17 patients)	
Varrato J. and Galetta S. [39]	2000	Hyperkinetic	Dystonia	Case report	1 patient with cervical torticollis	n = 1	65	n = 0	NA	Congenital fourth cranial nerve palsy unmasked by botulinum toxin injections	NA
Hong D. et al. [40]	2018	Hyperkinetic	Dystonia	Case series	2 siblings	n = 2	35 (case 1)	n = 1	NA	Mutations in the SYNJ1 gene	NA
							30 (case 2)				
Schieken R. et al. [41]	1972	Hyperkinetic	Chorea	Case series	2 patients with acute rheumatic fever	n = 2	7 (case 1)	n = 1	NA	Unilateral choreiform eye movements (transient)	NA
							14 (case 2)				
Giorelli M. et al. [45]	2013	Hyperkinetic	Tremor	Observational cross-sectional study	22 ET patients	0% (0 patients)	67.6 ± 9.1 (ET)	54.5% (n = 12), ET	NA	NA	Diplopia, frequent NMS in PD but not in ET
					31 PD patients	22.5% (7 patients)	69.1 ± 8.18 (PD)	70.9% (n = 22), PD			
Bryant J. et al. [46]	2003	Hyperkinetic	Tremor	Observational cross-sectional study	16 ET patients	n = 1	72.9	NA	22.8	Secondary to DBS of the ventro-intermedius nucleus of the thalamus	NA
Ackermans L. et al. [34]	2007	Hyperkinetic	Tourette syndrome	Case report	1 patient with Tourette syndrome	n = 1	39	n = 1	33	Vertical gaze palsy secondary to thalamic DBS	NA
Smith J. and Storey H. [35]	2019	Hyperkinetic	Stiff-person syndrome	Case report	1 patient with SPS	n = 1	57	n = 0	NA	Left inferior rectus weakness, skew deviation	NA
Piquet A. et al. [28]	2019	Hyperkinetic	Stiff-person syndrome	Prospective cohort study	17 patients with SPS	59% (n = 10)	Range from 17 to 75	NA	NA	Positive GAD65 antibodies in PSP patients with visual disturbances	NA
Mas N. et al. [42]	2015	Hyperkinetic	Stiff-person syndrome	Case series	3 patients with SPS	n = 1	33	n = 2	NA	Positive Glycine-receptor antibodies leading to diplopia and SPS symptoms	NA
Bernardo F. et al. [43]	2020	Hyperkinetic	Stiff-person syndrome	Case series	3 patients with SPS	n = 1	50	n = 3	NA	Positive anti-Zic4 antibodies leading to diplopia and SPS symptoms	NA
Kabanovski A. et al. [36]	2022	Hyperkinetic	Myoclonus	Case report	1 patient with myoclonus	n = 1	69	n = 1	NA	Skew deviation and oculopalatal myoclonus	NA
Danesh-Meyer H.V. [37]	2002	Hyperkinetic	Myoclonus	Case report	1 patient with myoclonus	n = 1	57	n = 0	NA	Skew deviation and oculopalatal myoclonus	NA

## Data Availability

The data presented in this study are available on reasonable request from the corresponding author.
